# Cook County Hospital

**Published:** 1871-03

**Authors:** Curtis T. Fenn

**Affiliations:** Chicago, Ill.


*Cook County Hospital.* Reported by CURTIS T. FENN, M.D., Chicago, Ill.

Nov. 22. Surgical. POWELL. Case I. Dislocation of the outer extremity of the clavicle—an exceedingly rare accident. Rheumatic patients sometimes had such displacements, but usually on both sides. A force capable of producing a dislocation like that would cause a fracture in ninety-nine cases out of a hundred. The extremity of this bone is movable. If rheumatism had been the cause, it would have been held firmly. The books stated that this dislocation was easily reduced, and held in place; but experience showed that little could be done. Shortening, from displacementor fracture of this bone, was of small moment. The indications for treatment were the same in dislocation and fracture. The old figure-of-eight bandage would be found to be the best, after a trial of all the other apparatuses for keeping the bone in place. In unruly patients and children, and in cold weather, the broad adhesive straps, first suggested by Prof. Freer, for such cases, ought to be used.

2d Case. Syphilitic disease of different types. Chancre nine months ago. A marked combination now of two or three different diseases, rupia being the principal. Syphilitic cachexia developed. The mucous membrane of the mouth covered with white patches; the skin white. The development was from the soft structures. It was secondary. He was anæmic, and exceedingly debilitated.

*Ques.* Was there anything having a tendency to increase the plasticity of the blood?

*Student.* Ans. Quinine and iron.

Yes; iodide of potassium would not do. We should select such preparations as could be taken. The elixirs of the shops contain little iron. Iron by hydrogen in pill answered exceedingly well; after a while iodide of potassium, with a tonic mixture. If it should be necessary, the following formula would do well: ℞. Pot. iod., ʒiv; Hyd. protiod., gr. iv; Aquæ, f℥iv—vj, M.

We should remember that the tendency of syphilitic disease was to cure itself. Such cases were not ordinarily received; but, if this patient was allowed to remain, he would be shown to illustrate the effect of tonic treatment in syphilis. Sleeping with that person might be productive of constitutional disease without the primary.

3d Case. Pistol shot wound of the chest. There was crackling in the cellular tissue; so the lung must have been wounded. On auscultation, bronchial breathing was discovered; so there must have been a little solidification. Inflammation had not been kept down by Norwood's tincture, tartarized antimony, or any such thing. The house surgeon had amused himself by poulticing the flesh wound.

4th Case. Necrosis of the spinal column. Disease traced to an injury. Matter had worked its way into the cavity of the chest. A pathological specimen of necrosis of the spinal column and angular or Pott's curvature; no lateral curvature; destruction of the body of one vertebra; processes not at all affected. The effort of repair often began by anchylosis of the transverse processes. The resulting paralysis was easily accounted for. The cord had been pressed upon; or, it had been bathed in pus and subjected to myelitis. The bodies of several vertebræ are usually eroded by pus. Nature effected a cure by anchylosis above and below. Treatment: Rest; taking the inflamed surfaces away from each other. There had been several such cases here. When post mortem examinations had been made, it had appeared perfectly surprising how such changes had been endured.

Pathological specimen. Sack of hydrocele. The testicle occupied the inferior and posterior portion of the sack. It was remarked, that we should not desire the obliteration of the sack, for the function of the testicle would be destroyed by this means. We should tap the sack low, passing the trochar upward, and avoiding the testicle; should allow the fluid to pass out, and should inject a gently stimulant fluid: the best was a solution of iodine, twenty grains to the fluid ounce. The tissues were offended by the injection of anything foreign, as warm water. Tinctures should not be used, for alcohol was a poison to the tissues. It was intended the next time such a case presented, to use the battery and electricity. On the sack being opened, fluid ran out, a little turbid. There was a beautiful, serous membrane, perfectly clear and clean. How to establish a rate of absorption equal to that of effusion, was the question.

Nov. 29. Medical. Ross. Pleurisy, with effusion and tuberculosis. Treatment: Poultice and small blisters, cod liver oil, and ferruginous tonics. The effusion would be absorbed better by such means at first, but after a while he should give fifteen grains of chlorate of potassa with the iron.

Surgical. POWELL. 1st Case. Club-foot. Subcutaneous division of the tendo Achillis several days ago. The patient not seen again till now. After the division, we should take hold of the foot and straighten it. A surgeon in Philadelphia broke the tendons and bones. We should keep the foot well in place, or the operation would amount to nothing. When the splint was applied the case was just begun. The object should be to get the patient to stepping on the sole of the foot; after that was attained, the case would improve constantly.

2d Case. Deep abscess of the lower jaw. There might be simply a collection of matter. A probe was passed in different directions, but did not come in contact with dead bone. It illustrated what should be done with deeply seated abscesses, whether lumbar, psoas, or simple. They should be opened freely at first, and afterwards treated antiseptically. As a consequence of neglect, here the matter had spread all over the cheek. We should examine this case again with interest. It would not do to poultice a thing like that too much.

3d Case. Epileptiform neuralgia, or tic douloureux of Trousseau. These cases formed a class of very great interest. There were two forms of the disease. 1. Non-convulsive. 2. Epileptiform. The first having no twitching; the second characterized by spasmodic movements of the muscles during the paroxysm of pain. Both forms always affected the terminal branches of the fifth pair of nerves only. The second branch, or submaxillary, was most frequently the seat of disease. He had first seen this case in 1867. It began in 1865. On entering the office, the man would sit down quietly, perhaps, then jump and grasp his head, and run around the room, apparently in great agony. The paroxysm would cease in a moment, when he would sit down again, and await its return sooner or later. If he talked or got cold, the fits became worse. They were aggravated by cold or damp weather. This was epileptiform neuralgia. The atrocious neuralgias, as Gross called them, were always epileptic. There is a disease called the tic, akin to St. Vitus' dance, in which the convulsion is in some of the terminal branches of the fifth, but without pain. In this case, the pain started from the region of the right mental foramen, and ran forward. All that was done then, was to divide the nerve at the mental foramen, and to tear out or exsect one inch of it. The pain ceased instantly, and stayed away for six months. This patient had previously received an incision from the temporal muscle to the lower part of the face, but it had done no good, for it had not touched the nerve. At the end of six months, he came back as he (Dr. P.) was going East. Probably, at that time, the jaw should have been trepanned, and the nerve still further removed. A little dental instrument should have been passed into the canal, and the nerve twisted out. But in the winter of 1867-8, a section of his jaw was removed. One year later this patient again returned. He went to the hospital and was operated on. The bone was found to be too friable for another resection, so the back portion of his jaw had to be disarticulated, and removed entire. Thereafter he remained free from pain one year and a half. But it has returned. Three weeks ago the pain came back severely, characterized by the former symptoms. He cannot describe it. (The patient being called on, stated he suffered so intensely at times, that his mouth became filled with blood without his being able to discover where it came from. At this point, a paroxysm occurred, apparently induced by mental excitement.) Only two things gave any prospect of relief: 1. Opium. 2. Division of the nerve. Trousseau, after a practice of over thirty years, said that he had never seen a case radically cured. But he was a physician. Perhaps the surgeon, by repeated exsection of the nerve, may effect a cure. It was the worst disease in existence. We should reflect on this case. The pain now had its seat in a fibrous band, taking the place of bone which was exsected, and in the end of the remaining jaw. The points to be remembered were, how to locate the disease, and to make a diagnosis. Then we should not be able to promise a great deal.

4th Case. Stump of leg. Re-amputation. Several causes. 1, projection of an angle of the tibia, making it difficult to adapt an artificial leg; 2, tissue drawn too tightly, increasing the danger of ulceration; and 3, dead bone. The operation should be performed as though it were the first. Assistants should always anticipate the wants of a surgeon. Chloroform administered.

The stump elevated to get as much blood back into the body as possible.

Dec. 2. Pathological. JOHNSON. Cirrhosis was not common in those who had hypertrophy or dilatation of the heart. Organs exhibited: *Heart*, weight, 14 oz. 5 dr.; *Spleen*, weight, 2 oz. 3 dr.; average weight, 7 oz., indicating a failure of nutrition. *Kidneys*, weight, 4½ oz.; normal weight, 4 to 6 oz. The surface is granular and mottled, the cortical portion reduced to a mere line in thickness. There was atrophy of the true secreting structure. The conducting structure normal. *Query.* Were the heart changes causes or effects? *Lungs.* The left adherent to the diaphragm, therefore there had been more or less of pleuritic inflammation. Certain conclusions might always be obtained during life. 1. Whether the heart was enlarged. 2. Whether hypertrophy preponderated. 3. Whether dilatation prevailed.

Surgical. POWELL. 1st Case. Fracture of the leg. Both bones united, though it had been slowly. The ankle was slightly stiff. If a starch bandage had been put on at first, the leg would have been concealed from view, and the ankle a good deal stiffer. A starch or plaster of Paris bandage should be applied, now, only to prevent accident and to encourage the patient to get out. He had been an extremely intemperate man, verging on delirium tremens when admitted. There had been no effort at repair of bone during the first four weeks.

2nd Case. Delirium tremens and fracture; admitted after a spree. Instead of trying to quiet by opium, they had sickened him by calomel and afterward tartarized antimony. This treatment was applied where opium had failed. Reference to the kind of splint needed. This person was very nervous, a mild form of the disease from an excess of stimulant. A little starch should be employed to prevent his getting the splint off. We should starch both the pad and the bands. All splints should be well padded. Simply rolling them with a bandage was not enough.

